# Lower birth weight and increased body fat at school age in children prenatally exposed to modern pesticides: a prospective study

**DOI:** 10.1186/1476-069X-10-79

**Published:** 2011-09-20

**Authors:** Christine Wohlfahrt-Veje, Katharina M Main, Ida M Schmidt, Malene Boas, Tina K Jensen, Philippe Grandjean, Niels E Skakkebæk, Helle R Andersen

**Affiliations:** 1 University Dept. of Growth and Reproduction, Rigshospitalet, Blegdamsvej 9, 2100 Copenhagen Ø, Denmark; 2 Institute of Public Health, Environmental Medicine, University of Southern Denmark, 5000 Odense, Denmark

**Keywords:** pesticides, prenatal exposure, birth weight, body composition, maternal smoking

## Abstract

**Background:**

Endocrine disrupting chemicals have been hypothesized to play a role in the obesity epidemic. Long-term effects of prenatal exposure to non-persistent pesticides on body composition have so far not been investigated. The purpose of this study was to assess possible effects of prenatal exposure to currently used pesticides on children's growth, endocrine and reproductive function.

**Methods:**

In a prospective study of 247 children born by women working in greenhouses in early pregnancy, 168 were categorized as prenatally exposed to pesticides. At three months (n = 203) and at 6 to11 years of age (n = 177) the children underwent a clinical examination and blood sampling for analysis of IGF-I, IGFBP3 and thyroid hormones. Body fat percentage at age 6 to11 years was calculated from skin fold measurements. Pesticide related associations were tested by linear multiple regression analysis, adjusting for relevant confounders.

**Results:**

Compared to unexposed children birth weight and weight for gestational age were lower in the highly exposed children: -173 g (-322; -23), -4.8% (-9.0; -0.7) and medium exposed children: -139 g (-272; -6), -3.6% (-7.2; -0.0). Exposed (medium and highly together) children had significantly larger increase in BMI Z-score (0.55 SD (95% CI: 0.1; 1.0) from birth to school age) and highly exposed children had 15.8% (0.2; 34.6) larger skin folds and higher body fat percentage compared to unexposed. If prenatally exposed to both pesticides and maternal smoking (any amount), the sum of four skin folds was 46.9% (95% CI: 8.1; 99.5) and body fat percentage 29.1% (95% CI: 3.0; 61.4) higher. There were subtle associations between exposure and TSH Z-score -0.66(-1.287; -0.022) and IGF-I Z-score (girls: -0.62(-1.0; -0.22), boys: 0.38(-0.03; 0.79)), but not IGFBP3.

**Conclusions:**

Occupational exposure to currently used pesticides may have adverse effects in spite of the added protection offered to pregnant women. Maternal exposure to combinations of modern, non-persistent pesticides during early pregnancy was associated with affected growth, both prenatally and postnatally. We found a biphasic association with lower weight at birth followed by increased body fat accumulation from birth to school age. We cannot rule out some residual confounding due to differences in social class, although this was adjusted for. Associations were stronger in highly exposed than in medium exposed children, and effects on body fat content at school age was potentiated by maternal smoking in pregnancy.

## Background

A dramatic increase in childhood overweight and obesity has been observed worldwide over recent decades. In addition to the psychosocial consequences, overweight children are at high risk of developing obesity in adulthood as well as cardiovascular and metabolic diseases [1]. There is accumulating evidence that the risk of adult chronic diseases is influenced by environmental factors acting *in utero *or during early childhood [2]. It has been suggested that suboptimal *in utero *environment leads to metabolic adaptations (so-called programming) in the foetus [3] which may increase susceptibility to future obesity [4]. An example of such an environmental factor is maternal smoking during pregnancy which is associated with both intrauterine growth restriction and increased risk of childhood overweight [5].

Environmental endocrine disrupting chemicals (EDCs) have been linked to development of overweight, especially if exposure occurs during pregnancy or early life [6-8]. Exposure to both persistent and non-persistent pesticides has been associated to reduced fetal growth e.g. birth weight and gestational age, but evidence is limited [9]. Furthermore, no prospective studies on long-term effects on body composition of currently used pesticides in humans have appeared.

We here report findings from a prospective study of a cohort of children born by women working in greenhouses during early pregnancy. The focus of this paper is the possible effects of prenatal exposure to currently used non-persistent pesticides on growth and body composition from birth to school age. We also report IGF-I and thyroid hormone levels, as these may be associated with obesity and exposure to EDCs [10,11].

## Methods

### Cohort and exposure assessment

In Denmark, all pregnant women, working in potentially hazardous job categories, are referred to departments of occupational medicine for risk assessment by their physician at the time of their first prenatal visit. From 1996 to 2000, pregnant women working in greenhouses and referred to the Department of Occupational Medicine in Odense, Denmark, were recruited consecutively to a prospective study evaluating effects of prenatal exposure to modern pesticides [12]. At enrollment, which typically occurred between 4 and 10 weeks of gestation, detailed information about working conditions, pesticide use and exposure was obtained from interviews of the women. Information about pesticide use was confirmed and supplemented by telephone contact to the employers. For all women, re-entry activities (such as moving or packing pot plants or nipping cuttings) constituted the main work functions. Approximately 20% of the women reported to have been directly involved in applying pesticides, mainly by irrigating fungicides or growth retardants. The women were categorized as occupationally exposed if pesticides were applied in the working area more than once a month and the women handled treated plants within one week after treatment and/or the women were directly involved in applying pesticides. The women were categorized as unexposed if none of the above criteria was fulfilled. Most of the women categorized as unexposed worked within the production of tomatoes, cucumbers or cactuses where chemical pesticides had been replaced with biological pest control or in separate greenhouses of other horticultures where pesticides were never or very seldom (once a month or less) used. The exposed women were furthermore categorized as medium or highly exposed as previously described [13]. Briefly, women who had direct contact to pesticides by applying, mixing or dipping cuttings in pesticides were rated as high or medium exposed depending on the exact procedure, the duration of the process, and the use of personal protective equipment. For re-entry activities, the exposure was rated as high if pesticides were used frequently (more than once a week) in the working area and the woman reported often to handle the treated plants without using gloves and to have had work functions with intensive plant contact. The exposure was rated as medium if pesticides were used infrequent, but the woman had intensive contact to the plants, or if pesticides were used frequently, but the women had no contact with the plant cultures within 24 hours after treatment. The exposure rating was done before the first examination of children by two toxicologists with special expertise in working conditions in greenhouse horticultures (independently, with agreement in all cases). The type and number of pesticides were not taken into consideration in this rating. Approximately 200 different pesticide formulations, representing 124 different active pesticide ingredients (11 growth regulators, 40 fungicides, 59 insecticides, and 11 herbicides), were used in the working areas. In general, the time elapse between pesticide treatment and handling of the plants (re-entry interval) was several days for the insecticides, but often only few hours for growth regulators and fungicides. Therefore, the main exposure for most of the women was assessed to be growth regulators and fungicides. The most used growth regulators were chlormequat chloride, daminozide, and paclobutrazol, and the most used fungicides were captan, chlorothalonil, iprodione, prochloraz, and propamocarb. The most used insecticides in the working areas were chlorpyrifos, fenazaquin, fipronil, methiocarb, methomyl, and pirimicarb. Although an increasing number of modern pesticides have been identified as potential endocrine disruptors in different in vitro assays [14-16], most of the active ingredients registered in this study have still not been investigated. Fourteen out of 21 pesticides, selected as the most frequently used in the working areas of the pregnant women, possessed endocrine disrupting potential in one or more cellular assays [15] thus indicating that a considerable part of the remaining pesticides may have similar properties. This is supported by a recent study in which nine pesticides not previously tested were identified as anti-androgenic [16]. Only very few modern pesticides have been specifically investigated for endocrine disrupting effects in animal studies but three of the fungicides used often in the greenhouses, fenarimol, vinclozolin, and prochloraz, have been demonstrated to be endocrine disruptors also in vivo [17-19]. In addition, low doses of some well-known neurotoxic organophosphate insecticides, as for example chlorpyriphos, during development caused excess weight gain in rats [20,21] maybe because of disturbances of the communication between adipose tissues and the brain [22]. A complete list of the pesticides used in the greenhouses can be obtained from the corresponding author. After the medical counseling and enrollment, pesticide exposed women were either moved to other work functions with none or minimal pesticide exposure, or went on paid leave. Hence the exposure classification of the women relates to the period before enrollment. Time from conception to removal from an exposed work situation was calculated for all exposed women. Pregnant women categorized as medium exposed worked median 38 days, (5-95 percentiles: 15-103 days) and women categorized as highly exposed worked median 30 days (5-95 percentiles: 18-48 days) after conception.

**Figure 1:**
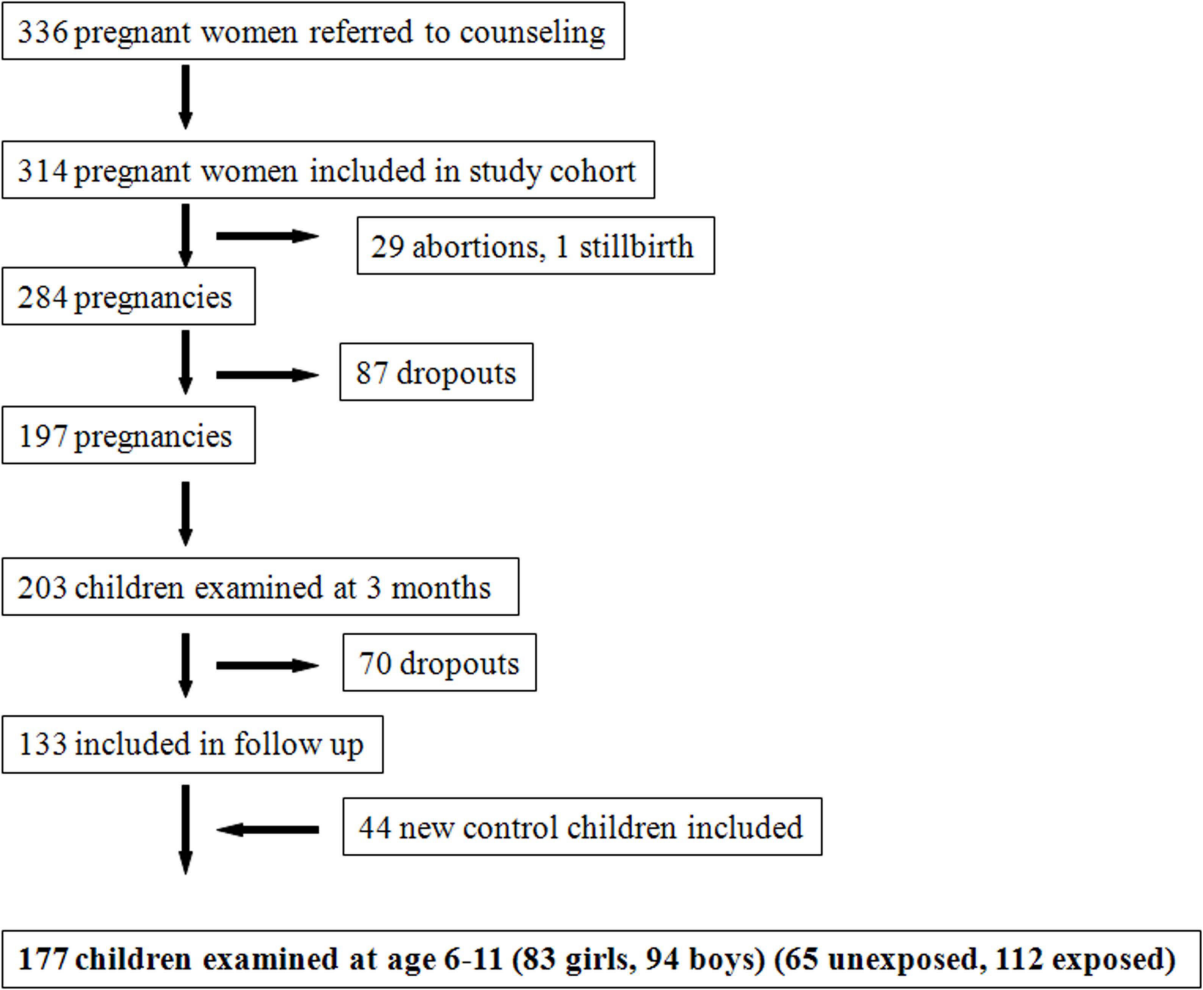
**Flowchart showing the numbers of included and dropouts from recruitment to examination**.

At 3 months of age, 203 children (113 boys, 90 girls) were examined (median age 3.1 months, 5-95 percentiles: 2.3; 4.4) [12]. A total of 168 children (91 boys, 77 girls) were categorized as prenatally exposed and 35 children (22 boys, 13 girls) as unexposed to pesticides. Of these children 133 (65.5%: 60% of unexposed and 66.6% of exposed children) accepted to participate in a follow-up study from 2007 to 2008 when they were between 6 and 11 years old (Figure 1). In order to increase the size of the unexposed control group, 44 children in the same age group were additionally recruited among families, friends and neighbours of the participants. None of the mothers to these children were occupationally exposed to pesticides during pregnancy.

### Clinical examination at school age

At follow-up the children underwent anthropometrical measurements and pubertal staging. Height was measured to the nearest 0.1 cm using a transportable stadiometer (Chasmors LTD., London, UK). Weight was measured on a digital weight scale with a precision of 0.1 kg (TBF-300, Tanita Europe, UK). Skin folds were measured at four sites (triceps, subscapular, flank/suprailiac and biceps) with a calliper (John Bull, British indicators LTD, UK) with a precision of 0.1 mm after allowing the jaws to close on the fat fold for two seconds [23]. All anthropometrical measurements were measured in triplicate and means were used for analysis. Pubertal staging [24,25] was performed by inspection and palpation of breast tissue (B) and by inspection for pubic hair (PH) in girls, and by evaluation of genital stage (G) and PH in boys. Puberty was dichotomized in girls as 0 for no sign of puberty (Tanner B1) and 1 for Tanner B2 or B3. In boys, 0 referred to no sign of puberty (Tanner G1), and 1 to Tanner G2. Eight boys did not consent to genital examination. The same pediatrician performed all clinical examinations (CWV), blinded to information about pesticide exposure. The variation in age at school-age follow-up is caused by the fact that the recruitment of pregnant women was done over four years (1996-2000), while the re-examination was conducted over only 6 months (2007- 2008).

### Questionnaire

Information about maternal lifestyle (including smoking, and alcohol during pregnancy) was obtained from a questionnaire in pregnancy [12] or, for newly included unexposed children, at enrollment. Information on birth weight and length was obtained from obstetric records.

Infant feeding pattern was coded as: no or little breastfeeding = 0, mainly or exclusively breastfeeding = 1, and alcohol consumption as no = 0, yes = 1. Information about maternal smoking in pregnancy (before enrollment) was evaluated (yes/no, mean number of cigarettes) and coded as no = 0, 1-9 cigarettes daily as 1, and 10 or more as 2.

At follow-up, all families completed a questionnaire on lifestyle and parental current work situation. Social class of the family (1 = high to 5 = low) was based on parental education and occupation according to National Standards [26]. The social class of the highest-ranking parent living with the child was used. Due to the small sample size, social class was categorized into group 1-3, group 4 or group 5. Group 4 was used as reference as most families belonged to this group.

### Blood samples and assays

Non-fasting peripheral venous blood samples were obtained (between midmorning and late afternoon) from 146 children at three months (116 exposed, 30 unexposed) and from 145 children at age 6-11 years (90 exposed, 55 unexposed). Serum concentrations of IGF-I and insulin-like growth factor binding protein 3 (IGFBP3) at 3 months were measured with a radio-immuno-assay (RIA) [27]. The limit of detection (LOD) was 21 ng/ml. IGFBP-3 was measured with RIA [28]. LOD was 300 ng/ml. At age 6-11 years, IGF-I and IGFBP3 were measured with solid-phase enzyme-labelled chemiluminescent immunometric assays (Immulite 2000; Diagnostic Products Corporation, Los Angeles, CA, USA) using World Health Organization (WHO) NIBSC IRR 87/518 and 93/560 standards, respectively. The limits of detection were 25 ng/ml and 500 ng/ml, respectively. Thyroid stimulating hormone (TSH) and thyroid hormones (thyroxine (T4); free T4; triiodothyronine (T3); and free T3) were measured with an electrochemiluminescense immunoassay (Modular Analytics E170, Roche GmbH, Mannheim, Germany).

### Statistics

Weight for gestational age (WGA) was expressed as the deviation (%) from the expected mean WGA [29]. Appropriate for gestational age (AGA) was defined as birth weight deviation between -22% and +22% which is equivalent to -2 and + 2 SD. Small for gestational age (SGA) was defined as WGA below -22% and large for gestational age (LGA) above 22%.

Body Mass Index (BMI) was calculated as kg/m^2 ^and Ponderal Index (PI) as kg/m^3^. Sum of four skinfolds (mm) was calculated as the sum of triceps + subscapular + biceps + flank skinfolds. Body fat percentage was calculated as suggested by Slaughter et al [30] by a equation using subscapular and triceps skinfolds: For girls: (1.33(triceps+subscapular) - 0.013(triceps + subscapular)^2 ^- 2.5) when sum of triceps and subscapular skinfold is < 35 mm and: (0.546(triceps+subscapular) + 9.7) when the sum is > 35 mm. For boys: (1.21(triceps+subscapular)- 0.008(triceps + subscapular)^2 ^-1.7) and: (0.783(triceps+subscapular) + 1.6), respectively.

Age- and gender-specific Z-scores were calculated (child value minus mean value for gender and age group divided by standard deviation for gender and age group) for BMI, thyroid hormones and IGF-I using contemporary Danish reference populations [31,32]. BMI Z-score difference (ΔBMI Z-score) from birth to school age was calculated by subtraction of Z-score at birth from Z-score at school age. A ΔBMI Z-score > 0.67 was considered a significant change (catch up or catch down), as 0.67 SD represents the width of each centile band of standard growth charts (i.e. 2^nd ^to 9^th^, 9^th ^to 25^th^, 25^th ^to 50 centiles etc.) [33]. Differences in ΔBMI Z-scores were evaluated as a categorical variable (+/- clinically significant change) with logistic regression analysis (adjusting for maternal smoking, social class and gestational age) and as a continuous variable with multiple linear regression analysis. The possible effects of prenatal pesticide exposure on birth outcomes and body composition at 6-11 years of age were analyzed by multiple linear regression analysis with exposure-, smoking- and social class- groups as dummy variables. Log-transformed variables were used when necessary to obtain normal distribution of residuals (sum of four skin folds, body fat percentage, hormones). Maternal smoking in pregnancy, maternal alcohol intake in pregnancy, smoking parents at any time in childhood, maternal ethnicity, breastfeeding status, social class, gender, multiple pregnancy, exact age at examination and gestational age were considered as possible confounders. Final models included variables that resulted in a change of estimates of more than 10%. IGF-I, IGFBP3, and thyroid hormones (in boys and girls separately) were adjusted for age, BMI, and puberty (in girls). IGF-I and TSH Z-scores were adjusted for BMI and puberty. Time of day of the blood sampling is known to affect serum levels of thyroid hormones [34,35], but was not included in the final model as it did not change the estimates.

Results are presented as the mean difference, or the relative difference in percent for log-transformed outcomes, with 95% confidence intervals (95%-CI) and significance levels.

### Ethics

The study was conducted according to the Helsinki II Declaration and approved by the Regional Danish Ethics Committee (S-20070068) and the Danish Data Protection Agency. Parents gave written informed consent.

## Results

Tables 1, 2, 3 provide population characteristics for all children included. Pesticide exposed children more often belonged to social group 4. There were no significant differences in age at examination, ethnicity, alcohol intake or smoking during pregnancy and breastfeeding between exposed and unexposed. However, within social group 1-3, 10% of the mothers smoked during pregnancy compared to 27% in social group 4 and 43% in group 5. Triplets and twins were only found among exposed and thus removed from analyses of birth outcomes.

**Table 1:** Population and birth characteristics† for all children (n = 247) in the Danish greenhouse cohort 1996-2000

	Girls		Boys	
	
	Unexposed (n = 36)	Pesticide exposed (n = 77)	Unexposed (n = 43)	Pesticide exposed (n = 91)
Birth weight (g) *	3640 (2842; 4600)	3440 (2300; 4200)	3800 (2850; 4720)	3525 (2321; 4400)

Gestational age (days)	282 (262; 294)	282 (260; 298)	282 (263; 297)	281 (252; 296)

Twin or triplet pregnancy *	0 (0)	4 (5.2)	0 (0)	7 (7.7)

Small for gestational age *	1 (2.8)	5 (6.5)	0 (0)	7 (7.7)

Large for gestational age	3 (8.3)	1(1.3)	3 (7.0))	1 (1.1)

Weight for gestational age (% deviation)*	2(-17; 24)	-3(-26; 18)	1(-18; 22)	-4(-27; 19)

Birth length (cm)	52(49; 58)	52(48; 56)	53(49; 56)	53(48; 56)

BMI (kg/m^2^) *	13.2(11.4; 16.4)	12.8(10.0; 15.0)	13.1(11.3; 15.2)	13.0(10.4; 15.0)

Ponderal index (kg/m^3^) *	25.3(21.4; 28.6)	24.2(20.2; 29.8)	24.8(22.4; 28.1)	24.5(20.2; 28.1)

Smoking in pregnancy (yes)	11 (32.4)	20 (26.0)	12 (27.9)	29 (32.6)

Alcohol intake in pregnancy‡	12 (41.4)	19 (36.5)	9 (25.7)	23 (39.7)

Breastfeeding at 3 months§	8(61.5)	54 (70.1)	13 (61.9)	64 (72.7)

* p < 0.05. Difference between exposed and unexposed (boys and girls together) tested with Fisher's exact test for categorical variables and Mann-Whitney U-test for continuous variables.

†Values are presented as n (%) (categorical variables) or median (5; 95 percentiles) (continuous variables). Information from new controls was collected from birth records and questionnaire at school age (smoking and alcohol).

‡ Information on alcohol intake (yes/no) (n = 174).

§ Information on breastfeeding until 3 months (mainly or exclusively/little or no), (n = 200: 13 unexposed girls, 21 unexposed boys).

**Table 2:** Characteristics of 203 children examined at 3 months of age†

	Girls		Boys	
	
	Unexposed (n = 13)	Pesticide exposed (n = 77)	Unexposed (n = 22)	Pesticide exposed (n = 91)
Age (months)	3.0(2.4; 4.0)	3.1(2.3; 4.4)	3.1(2.3; 3.8)	3.1(2.3; 4.5)

Weight (g) at 3 months	6230(5160; 7240)	5910 (5050; 7150)	6810 (5490; 7790)	6640 (5510; 7980)

Weight gain from birth to 3 months	2480(1780; 3240)	2400(1400; 4120)	2930(2300; 4180)	2990(1870; 4800)

Length at 3 months (cm)	60.9(57.6; 64.5)	60.2(57.5; 63.8)	62.0(60.2;65.3)	61.9(58.2; 66.2)

BMI at 3 months (kg/m^2^)	16.3(15.1; 19.5)	16.3(14.2; 18.7)	17.4(14.9; 20.7)	17.2(15.6; 20.0)

BMI Z-score at 3 months (SD)	-0.01(-0.75; 2.02)	-0.02(-1.35; 1.52)	0.38(-1.07; 2.29)	0.27(-0.64; 1.89)

Δ BMI Z-score since birth	-0.67(-2.03; 0.98)	-0.14(-2.29; 3.35)	0.24(-1.37; 1.52)	-0.06(-1.64; 2.58)

†Values are presented as n (%) (categorical variables) and median (5; 95 percentiles) (continuous variables)

No significant differences between exposed and unexposed (boys and girls together or separately) tested with Mann-Whitney U-test.

**Table 3:** Characteristics of 177 children examined at 6-11 years of age†

	Girls		Boys	
	
	Unexposed (n = 30)	Pesticide exposed (n = 53)	Unexposed (n = 35)	Pesticide exposed (n = 59)
Social group 1-3 *	13 (43.3)	12 (22.6)	13 (37.1)	11 (18.6)

Social group 4 *	10 (33.3)	31 (58.5)	15 (42.9)	32 (54.2)

Social group 5	7 (23.3)	10 (18.9)	7 (20.0)	16 (27.1)

Maternal origin other than Danish	1 (3.3)	1 (1.9)	3 (8.6)	3 (5.1)

Age at examination (years)	8.05 (6.65-10.78)	8.82 (6.78-10.16)	8.71 (6.39-11.01)	8.39 (6.98-10.32)

In puberty at examination	7(23.3)	24(45.2)	3(9.4) (n = 32‡)	0 (n = 54‡)

BMI (kg/m^2^)	16.3(13.8-21)	16.2(13.9-22.4)	15.7(14.3-19.7)	16.8(14-21.8)

BMI Z-score (SD)	0.28(-1.03-3.15)	0.19(-0.91-3.97)	-0.1(-1.09-2.84)	0.45(-1.27-3.29)

Δ BMI Z-score since birth (SD) *	-0.55(-1.87; 2.59)	0.53(-2.06; 4.66)	-0.21(-2.52; 3.11)	0.54(-2.19; 3.67)

Sum of four skinfolds (mm)*	42.10(27.5-82.4)	45.3(28.8-108.9)	33.4(19.4-82.6)	39.1(23-86.6)

Body fat percentage (%) *	18.53(12.99-31.32)	19.14(13.57-37.82)	16.56(10.12-30.41)	18.40(11.36-32.22)

* p < 0.05. Difference between exposed and unexposed (boys and girls together) tested with Fisher's exact test for categorical variables and Mann-Whitney U-test for continuous variables.

†Values are presented as n (%) (categorical variables) and median (5; 95 percentiles) (continuous variables)

‡Puberty staging could not be obtained in eight boys.

Birth weight and WGA was significantly lower in both medium and highly exposed children, also after adjustments for covariates (table 4). For all birth outcomes effect estimates were stronger in highly exposed than medium exposed. Birth length, BMI, and PI were lower in the exposed group but only statistically significant for BMI in highly exposed. Gestational age was not significantly different between groups (table 1). Including twins and triplets did not change estimates significantly. Birth outcomes did not differ between subjects followed to school age compared to those that were not. At three months (table 2) there were no significant differences between weight, height, weight gain since birth, BMI, BMI Z-score, or ΔBMI Z-score in exposed and unexposed.

**Table 4:** Results from multiple linear regression analysis† for birth outcomes and relevant covariates

Covariates in model	Weight for gestational age (%)	Birth weight (g)	Birth length (cm)	Body mass index (kg/m^2^)	Ponderal index (kg/m^3^)
Gestational age (days)	-------	27(22; 32)*	0.1(0.1; 0.1) *	0.05(0.03; 0.06) *	0.04(0.01; 0.07)*

Female gender	-------	-132(-247; -18) *	-0.8(-1.4; -0.3)*	-0.1(-0.4; 0.2)	0.3(-0.3; 0.9)

Maternal smoking in pregnancy < 10 cigarettes	-2.2(-7.0; 2.6)	-77(-251; -97)	-0.3(-1.0; -0.5)	-0.2(-0.6; 0.2)	-0.3(-1.2; 0.6)

Maternal smoking in pregnancy ≥ 10 cigarettes	-6.0(-10.3; -1.6) *	-213(-372; -54) *	-0.7(-1.0; -0.0) *	-0.4(-0.8; -0.0)*	-0.5(-1.3; 0.3)

Prenatal pesticide exposure medium	-3.6(-7.2; -0.0) *	-139(-272; -6) *	-0.3(-1.0; 0.3)	-0.2(-0.6; 0.1)	-0.3(-1.0; 0.4)

Prenatal pesticide exposure high	-4.8(-9.0; -0.7) *	-173(-322; -23) *	-0.4(-1.0; 0.3)	-0.4(-0.8; -0.0) *	-0.7(-1.4; 0.1)

* p < 0.05. † n = 236 (247-11 children from multiple pregnancies). Results are expressed as mean differences (95%-CI)

Example: Birth weight is 173 g lower in highly exposed children compared to unexposed, when correcting for gestational age, female gender and maternal smoking level in pregnancy

The exposed children had a larger increase in BMI Z-score from birth to school age (mean difference: 0.55 (95% CI: 0.1; 1.0)) than unexposed children. Likewise exposed children had 2.2 (95% CI: 1.0; 4.5) times higher odds ratio (OR) of an increase in ΔBMI Z-score > 0.67.

Measures of body composition (individual skin folds (data not shown), sum of four skin folds, body fat %, BMI, and BMI Z-score) were all higher in the exposed group (except BMI and BMI Z-score in girls) (table 3). Statistical significance after adjustment for confounders was reached for flank skin fold (data not shown), and for those highly exposed for triceps skinfolds (data not shown), sum of four skinfolds and body fat percentage (table 5). When data analyses were repeated (same models) for the two groups of unexposed children separately, the trends were the same but the results were not significant. Difference in birth weight (estimate in g (95% CI)) for unexposed with greenhouse working mothers versus medium exposed was -117(-284; 50), for unexposed with greenhouse working mothers versus highly exposed: -142(-319; 36), for new children versus medium exposed: -214 (-378; -49), and for new children (not greenhouse working mothers) versus highly exposed: -235(-413; -57). Likewise estimates for body fat percent (percent difference in body fat percent (95% CI)) for unexposed greenhouse working mothers versus medium exposed was 3.0(-11.7; 20.5), for unexposed greenhouse working mothers versus highly exposed: 10.2(-6.5; 30.0), for new children versus medium exposed: 6.7(-5.5; 20.2), and for new children versus highly exposed: 13.8(0.0; 29.4). When including only prepubertal children in the analyses, the estimates did not change appreciably (e.g. ΔBMI Z-score Β (95% CI): medium: 0.13(-.36; 0.63) high: 1.0 (0.43; 1.58).

**Table 5:** Results from multiple regression analysis of body composition outcomes and relevant covariates in children at 6-11 years of age†

Outcome	Β (95% CI)	p
Covariates in model		

**BMI Z-score**		

Social class 5 ‡	0.04(-0.45; 0.53)	0.876

Social class 1-3 ‡	-0.54(-1.03; -0.09)	0.023

Maternal smoking in pregnancy (< 10 cigarettes)	0.68(0.09; 1.29)	0.025

Maternal smoking in pregnancy (≥ 10 cigarettes)	0.48(-0.10; 1.05)	0.106

Maternal pesticide exposure medium level	0.17(-0.28; 0.63)	0.452

Maternal pesticide exposure high level	0.36(-0.13; 0.86)	0.150

**ΔBMI Z-score between school age and birth**		

Gestational age (days)	-0.06(-0.08; -0.04)	0.000

Social class 5 ‡	0.28(-0.28; 0.82)	0.324

Social class 1-3 ‡	-0.75(-1.3; -0.22)	0.006

Maternal smoking in pregnancy (< 10 cigarettes)	0.76(0.11; 1.44)	0.025

Maternal smoking in pregnancy (≥ 10 cigarettes)	0.76(0.11; 1.41)	0.022

Maternal pesticide exposure medium level	0.49(-0.02; 0.99)	0.061

Maternal pesticide exposure high level	0.63(0.08; 1.19)	0.026

**Sum of four skin folds**, mm (log transformed)		

Female gender	22.2(8.4; 37.4)	0.01

Age, years	4.7 (-0.2; 9.9)	0.057

Social class 5 ‡	1.4(-12.7; 17.5)	0.876

Social class 1-3 ‡	-14.7(-25.5; -0.9)	0.030

Maternal smoking in pregnancy (< 10 cigarettes)	13.5(-5.2; 36.1)	0.165

Maternal smoking in pregnancy (≥ 10 cigarettes)	16.9(1.8; 39.6)	0.078

Maternal pesticide exposure medium level	9.4(-4.5; 25.3)	0.198

Maternal pesticide exposure high level	15.8(0.2; 34.6)	0.053

**Body fat percentages (log transformed)**		

Female gender	12.5(2.8; 22.7)	0.009

Age, years	3.3(0.5; 7.2)	0.096

Social class 5 ‡	-1.1(-11,7; 10.9)	0.860

Social class 1-3 ‡	-10.1(-18.9; 2.3)	0.067

Maternal smoking in pregnancy (< 10 cigarettes)	8.4(-5.6; 24.5)	0.232

Maternal smoking in pregnancy (≥ 10 cigarettes)	14.5(2.3; 31.2)	0.026

Maternal pesticide exposure medium level	5.7(-4.9; 17.2)	0.277

Maternal pesticide exposure high level	13.0(0.7; 26.8)	0.034

† Expressed as differences (95%-CI) for BMI Z-score and ΔBMI Z-score between school age and birth, and relative differences in percent (95%-CI) for Sum of four skin folds and body fat percentage (log-transformed variables). ‡Social group 4 is used as reference.

Children prenatally exposed to both pesticides and maternal smoking in pregnancy (any amount) had higher sum of four skin folds (46.9% 95% CI: 8.1; 99.5, p = 0.015) and body fat percentage (29.1%, 95% CI: 3.0; 61.4, p = 0.028) (adjusted for age, sex and social group) (statistically significant interactions between exposure and maternal smoking in pregnancy: p = 0.009 and p = 0.003)

IGF-I and IGFBP3 concentrations at three months of age did not significantly differ between exposed and unexposed children (table 6). At 6-11 years of age, exposed girls had lower IGF-I levels (-24% [95%-CI: -44.5; -0.6], p = 0.008) and IGF-I Z-scores (-0.62(-1.0; -0.22), p = 0.003) than unexposed girls (but no difference between medium and highly exposed children). In boys there was a tendency towards higher levels of IGF-I and IGF-I Z-scores in the exposed compared to unexposed, significant only for the highly exposed (IGF-I Z-score: 0.38 (-0.03; 0.79), medium exposed: 0.21 (-0.23; 0.65), highly exposed: 0.69 (0.18; 1.20). IGFBP-3 levels did not differ between the groups (table 6).

**Table 6:** Serum concentrations of IGF-I, IGF-BP3, and thyroid hormones in children in the Danish greenhouse cohort (medians (5; 95 percentiles))

	Girls		Boys	
	**Unexposed**	**Pesticide exposed**	**Unexposed**	**Pesticide exposed**

IGF-I (3 months †) (ng/ml)	76(55; 116)	81(34; 121)	97(45; 161)	92(57; 134)

IGFBP3 (3 months†) (ng/ml)	1442(1269; 2080)	1824(1247; 2444)	1868(1162; 2830)	1866(1288; 2472)

IGF-I (6-11 years ‡) (ng/ml)	188(101; 315)	157(75; 241)	118(60; 214)	151(69; 238)

IGFBP3 (6-11 years ‡)(ng/ml)	3550(2360; 4840)	3490(2440; 4960)	3150(2410; 4140)	3460(2240; 4740)

TSH (6-11 years ‡) (mU/l)	2.40(0.83; 4.79)	2.03(0.99; 4.19)	2.28(1.23; 5.60)	2.13(1.03; 4.31)

T3 (6-11 years ‡) (nmol/l)	2.63(2.19; 3.15)	2.49(1.99; 3.50)	2.38(1.97; 2.86)	2.49(2.15; 3.28)

T4 (6-11 years ‡) (nmol/l)	117.5(105.8; 148.0)	112.0(87.1; 157.3)	107.6(84.1; 140.9)	107.2(82.70; 115.5)

Free T3 (6-11 years ‡) (pmol/l)	6.78(5.56; 7.67)	6.43(4.87; 7.78)	6.37(5.29; 7.36)	6.58(5.60; 7.57)

Free T4 (6-11 years ‡) (pmol/l)	18.0(16.2; 20.3)	17.4(14.3; 21.6)	17.9(15.8; 22.1)	17.3(14.7; 22.1)

† At 3 months n = 146; boys: 67 exposed, 16 unexposed, girls: 49 exposed, 12 unexposed

‡ At age 6-11 years n = 145; boys: 53 exposed, 31 unexposed, girls: 37 exposed, 24 unexposed

TSH Z-scores at school age (table 6) were non-significantly lower in exposed compared to unexposed children (B: -0.485 [95% CI: -1.132; 0.162], p = 0.141) (no gender difference). When adjusting for BMI, the differences became significant -0.655 [-1.287; -0.022], p = 0.043, though without a trend of stronger association in highly exposed. We found no differences related to pesticide exposure in peripheral thyroid hormone concentrations.

## Discussion

In this longitudinal study of children born by women working in greenhouses during pregnancy, we found several indices that prenatal exposure to modern pesticides can have both immediate and long term adverse effects on body composition. More than 100 pesticides with several possible mechanisms of action were used in the greenhouses. Thus, effects cannot be ascribed to any individual substances but may be related to the combined exposure to several pesticides (and "inert" ingredients in their formulations). Although fetal exposure to pesticides was only brief during early pregnancy and the used pesticides were non-persistent, we found significantly lower birth weight and weight for gestational age in exposed children, but no effect on gestational age or on growth parameters at 3 months. At school age, the exposed children had gained thicker skin folds and a higher body fat %, and this effect was augmented if the children were simultaneously exposed to maternal smoking. Stronger associations were found in children with highly pesticide exposed mothers compared to those with medium exposed mothers for all growth outcomes. The possible bi-phasic effects of pesticides on prenatal versus postnatal growth were visible due to the longitudinal design of this study, which allowed the calculation of changes in age and gender adjusted BMI scores over time. Absolute measures such as body weight, height or BMI at school age were less clearly associated with pesticide exposure. Thus, the here-described observations could have been missed in cross-sectional studies.

Along with the changes in body composition, we found subtle but significant differences in IGF-I and TSH Z-score between the exposed and unexposed children. IGF-I plays an important role in insulin sensitivity and glucose homeostasis in children [36]. In adults, low serum IGF-I concentrations predict a higher risk of progression to impaired glucose tolerance and type 2 diabetes [37]. The observed effects of pesticides on IGF-I concentrations appeared to be sex specific, i.e. lower IGF-I in exposed girls and a tendency to higher IGF-I in exposed boys. Sex-dimorphic effects on IGF-I levels have also been reported for DDE (dichlorodiphenyldichloroethylene) [38].

Recent human studies found that prenatal exposure to persistent pesticides was associated with increased childhood BMI [7,39,40]. One study [41] addressed the issue of potential interactions between smoking and chemical exposures during pregnancy on the growth of children. In line with our findings (with non-persistent pesticides), they reported that DDE in cord blood correlated positively with BMI at 3 years, and that this effect was enhanced by maternal smoking. Few studies have addressed effects of currently used *non-persistent *pesticides on human growth. Studies in rats have shown late effects on weight and body size of male rats prenatally exposed to the non-persistent organophosphate insecticide chlorpyrifos [20]. Other studies have indicated that some non-persistent pesticides may have effects on thyroid function. Prenatal exposure to the fungicide vinclozolin caused lower TSH and T4 serum concentrations in rats [42]. Prenatal exposure to the fungicide prochloraz reduced the serum concentration of T3, but not TSH and T4, in male pups [43].

Two human studies have associated non-persistent pesticide use with low birth weight [44,45]. After residential use of chlorpyrifos in New York City, the concentration in cord blood was inversely associated with birth weight and birth length [46]. In contrast, a study among women in an agricultural community found that exposure to organophosphate pesticides resulted in lower gestational age, but had no effect on birth weight [47]. None of the studies of non-persistent pesticides have investigated long-term effects on growth or body composition.

The longitudinal design is an important strength of our study. The evaluation of mothers as medium/highly exposed or unexposed was done in early pregnancy completely blinded to subsequent child outcomes and is to our best belief a valid classification. All examinations and analyses of serum samples were likewise done blinded to exposure information and birth outcomes. Birth data were retrieved from obstetric records, thereby reducing the risk of information bias. Information on smoking in pregnancy was obtained during pregnancy at the time of enrolment in the original cohort, whereas those that participated only in the follow up answered retrospectively for the entire pregnancy. If recall bias was introduced here, it is most likely to have caused an underestimation of maternal smoking in pregnancy for these children. Follow-up time varied from 6 to 11 years, but this was taken into account by adjusting for age and using sex and age specific Z-scores when possible.

More than 30% of the children were lost to follow up at school age, introducing a possibility for selection bias. Reasons for dropout could be that obese children would be less interested in attending a clinical examination, and mothers knowing they had been exposed may be more inclined to stay in the study. However, dropout rates were comparable and birth outcomes of the children lost to follow did not differ from the children in the study. New unexposed children were recruited at school age to gain statistical power which introduced small differences in social class between exposed and unexposed, hereby again introducing possible selection bias. We therefore performed analyses comparing the exposed mother to unexposed mothers either working in greenhouses or not. The trend was the same, but estimates were as expected stronger for the larger group of children of non-greenhouse working mothers.

Social class is known to play a significant role in childhood obesity, an effect, which was also seen in our study population, and we cannot rule out some residual confounding due to differences in social class between exposed and unexposed children. We did however adjust for this confounder in our statistical analyses as well as for maternal smoking, but this did not remove the observed pesticide-related associations. We cannot exclude that the findings of associations between TSH Z-score and IGF-I levels in relation to exposure are chance findings or secondary to the changes in body composition. We did not obtain blood samples from all children, and this attrition may have reduced the statistical power to detect all relevant associations.

Although prenatal pesticide exposure appears to precipitate pubertal development in girls [Wohlfahrt-Veje C et al., manuscript in preparation], we found that effect estimates were not markedly different if based only on prepubertal children. Adjustment for pubertal stage would therefore not be appropriate, when the prenatal pesticide exposure may have affected both body fat and onset of puberty. Similar considerations were made in a study of possible effects of DDE on female offspring of Michigan anglers and fish eaters, where DDE affected age at menarche as well as body size [48].

## Conclusions

Occupational exposure to currently used pesticides may have adverse effects in spite of the added protection offered to pregnant women. Our study showed that maternal exposure to combinations of modern, non-persistent pesticides during early pregnancy may cause long-term effects in offspring. We cannot rule out some residual confounding due to differences in social class, although this was adjusted for. We found that higher level of maternal exposure in early pregnancy was associated with lower child weight at birth, followed by increased body fat accumulation from birth to school age. Maternal smoking in pregnancy potentiated the associations to body fat accumulation.

## Abbreviations

BMI: Body mass index; PI: Ponderal index; WGA: Weight for gestational age; SGA: Small for gestational age; AGA: Appropriate for gestational age; LGA: Large for gestational age; T3: Triiodothyronine; FT4: Thyroxine; TSH: Thyroid stimulating hormone; IGFBP3: Insulin-like growth factor binding protein 3; LOD: Limit of detection

## Competing interests

The authors declare that they have no competing interests.

## Authors' contributions

CWV contributed to conception and design of the follow up study, examined all the children at the follow up, and was the main person in interpretation, analysis and writing of the manuscript. KMM contributed to conception and design of both the infant- and the follow-up- study and gave substantial contributions to analysis, interpretation and revision of the manuscript. IMS contributed to conception and design of infant study, examined all the infants and gave substantial contributions to analysis and revision of the manuscript. MBO examined the children that were used for reference material and gave contributions to analysis and revision of the manuscript. TKJ contributed to conception and design of the follow up study and gave substantial contributions to analysis, interpretation and revision of the manuscript. PG contributed to conception and design of both the infant- and the follow-up- study and gave contributions to analysis, interpretation and revision of the manuscript. NES contributed to conception and design of both the infant- and the follow-up- study and gave contributions to analysis, interpretation and revision of the manuscript. HRA was the main contributor in conception and design of both the infant- and the follow-up- study and gave substantial contributions to analysis, interpretation and revision of the manuscript.

All authors read and approved the final manuscript
